# Perception of climate change and its impact by smallholders in pastoral/agropastoral systems of Borana, South Ethiopia

**DOI:** 10.1186/s40064-015-1012-9

**Published:** 2015-05-20

**Authors:** Nega Debela, Caroline Mohammed, Kerry Bridle, Ross Corkrey, David McNeil

**Affiliations:** Tasmanian Institute of Agriculture, University of Tasmania, Hobart, TAS 7001 Australia

**Keywords:** Adaptation, Farm households, Pastoral/agropastoral systems, Perception, Rainfed agriculture, Vulnerability

## Abstract

This study investigates the perception of historic changes in climate and associated impact on local agriculture among smallholders in pastoral/agropastoral systems of Borana in southern Ethiopia. We drew on empirical data obtained from farm household surveys conducted in 5 districts, 20 pastoral/agropastoral associations and 480 farm households. Using this data, this study analyses smallholders’ perception of climate change and its associated impact on local agriculture, and the effect of various household and farm attributes on perception. Results suggest that most participants perceived climatic change and its negative impact on agricultural and considered climate change as a salient risk to their future livelihoods and economic development. Different levels of perception were expressed in terms of climate change and the impact on traditional rain-fed agriculture. Age, education level, livestock holding, access to climate information and extension services significantly affected perception levels. Household size, production system, farm and non-farm incomes did not significantly affect perception levels of smallholders. Smallholders attributed climate change to a range of biophysical, deistic and anthropogenic causes. Increased access to agricultural support services, which improves the availability and the quality of relevant climate information will further enhance awareness of climate change within of the rural community and result in better management of climate-induced risks in these vulnerable agricultural systems.

## Introduction

Perception strongly affects how farmers deal with climate-induced risks and opportunities, and the precise nature of their behavioural responses to this perception will shape adaptation options, the process involved and adaptation outcomes (Adger et al. 2009; Pauw 2013). Misconception about climate change and its associated risk may result in no adaptation or maladaptation thus increasing the negative impact of climate change (Grothmann and Patt 2005).

Rural households in sub-Saharan Africa are heavily reliant on their natural resource base to provide food and income for the family, and the availability of such resources is dependent on favourable seasonal weather conditions (Solomon et al. 2007). In the climatically more variable regions of sub-Saharan Africa, where dryland farming systems are common, the heavy reliance on rainfed agriculture increases the vulnerability of rural households to the adverse impacts of climate change (Thomas et al. 2007; Mertz et al. 2009). Resource-poor farmers have limited capacity to adapt and are particularly vulnerable (Antwi-Agyei et al. 2012). In Ethiopia, agricultural production is predominantly rain-fed and irrigated agriculture constitutes only 1.1 % of the total cultivated land (Bewket and Conway 2007) and less than 3 % of the current food production in the country (Awulachew et al. 2005). Pastoralism in Ethiopia represents about 60 % of the land mass and much of the commercially valuable livestock is produced under rainfed dryland small-scale agricultural systems vulnerable to the adverse impacts of climate change (Little et al. 2010; Fratkin 2014). In addition to climate change agricultural systems in developing countries are faced with other risks such as demand for rapidly growing population, changing land tenure systems and ecological degradation (Jones and Thornton 2009; Rufino et al. 2013).

The current international scientific consensus is that recent global warming conditions indicate a fairly stable long-term trend with natural variability of local climate (Hansen et al. 2012). The notorious variability in local climate conditions and the underlying long-term trend towards global warming makes it difficult for local people to discern climate change. Beliefs and attitudes towards climate change depend on contextual factors including access to climate information and experiential learning. For instance, the large majority of scientists working in disciplines contributing to studies of our climate, accept that climate change is almost certainly being caused by human activities (Hansen et al. 2012). Indigenous people with limited access to climate information are more likely to attribute changing climatic conditions, particularly extreme weather events, to a change in their rituals and cultural practices (Nyanga et al. 2011). Irrespective of the driving forces however understanding views of target communities is important to prompt the need to adapt and facilitate support for policy related adaptation decisions.

Perception of climate change among rural communities is driven by multiple forces. Different household and farm factors influence whether and to what extent farmers perceive climate change and its impact on local agriculture (Deressa et al. 2011). The age of a subsistence farmer is closely related to farming experience and their accumulated knowledge of the environment including changes in climatic conditions (Patt and Schröter 2008; Deressa et al. 2011; Juana et al. 2013) that may go back many decades. Studies conducted in African smallholder farming systems have indicated that the level of formal education attained by farmers influences their ability to perceive climate change and its impact (Maddison 2007; Mustapha et al. 2012). Households with many members are more likely to engage in non-farm income generating activities because non-farm income buffers financial losses from farming, the householders are less likely to perceive climate change (Ndambiri et al. 2012). Access to support services such as extension services and climate information is purported to increase farmer perception of climate change and its associated risks (Maddison, 2007, ATPS, 2013).

Livestock ownership and herd size in traditional farming systems are two related variables which have been used to represent the level of a farmer’s dependence on natural resources such as pasture and water for extensive livestock production (Kemausuor et al. 2011; Legesse et al. 2013). The availability of such natural resources depends on a combination of resource management strategies and climatic conditions. Different livestock groups in this regard have varying degrees of susceptibility to stress conditions such as more frequent and longer periods of drought under a changing climate. For instance, cattle known for slower biological turnover are considered more vulnerable to feed shortages during drought than small ruminants and camels (Lesnoff et al. 2012). Households with cattle, in the event of drought, carry a potentially diversifiable risk (idiosyncratic risk) as well as the aggregate or co-variant risk of drought at a regional scale (Ligon and Schechter 2003; Lesnoff et al. 2012). However, larger herd size is associated with greater demand for food.

This study uses a psychometric approach to explore how and why the traditional smallholders in the Ethiopian Borana pastoral/agropastoral systems have perceived changes to climate over a 20-year period (1992–2012). Psychometrics is one approach commonly applied to the study of perception in different disciplines including climate change (Sjöberg 2000a). Combined with meteorological evidence from nearby stations, psychometric modelling can be used to generate useful policy-relevant information to better understand the extent of perception by farming communities (Maddison 2007; Deressa et al. 2011; Legesse et al. 2013). Studies in climate change perception using psychometric modelling often generate information aimed at better understanding the extent of farmer perception, but fail to identify the factors that influence the level at which smallholders perceive climate change and its impact. In addition, much more emphasis is given to the mainstream sedentary agricultural systems with less attention to more marginalized pastoral/agropastoral systems. This study therefore examines smallholders’ perception of climate change and its impact on agriculture in the pastoral/agropastoral systems of Borana vulnerable to climate change. Our results improve our knowledge of smallholder perception in the Borana traditional system and can be used by decision makers seeking to improve adaptation processes and outcomes.

## Methods

### The study area

**Fig. 1:**
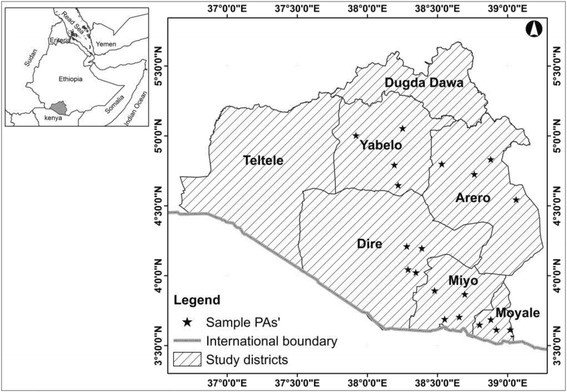
Map of the study area, Borana pastoral/agropastoral systems, Southern Ethiopia (Note: PA = Pastoralist/agropastoralist association)

The study area, Borana pastoral/agropastoral systems lie within the Borana administrative zone (3°36’ and 6°38’N and 36°43’ and 41°40’E) which is located in southern Ethiopia in the tropics and shares boundary with Northern Kenya in the south (Fig. 1). The Borana administrative zone is broadly divided into two agroecological zones - the high-altitude humid lands to the north and semi-arid lowlands to the south (Tache and Irwin 2003). The study was carried out in the heartland of (agro-)pastoral farming systems, in five of the seven semi-arid lowland administrative districts of the Borana Plateau (Yabelo, Dire, Moyale, Miyo, Arero, Teltele and Dugda Dawa). The study area has two dry seasons and two rainy seasons; *Bona* (long dry spell from December to February), *Gana* (long rainy period from March to May), *Adolessa* (short dry spell from June to August) and *Hagaya* (short rainy period from September to November) with remarkable inter-annual variation in seasonal conditions (Tache and Irwin 2003).

### The study approach

The study focuses on perception of climate change by farm households over a twenty-year period (1992–2012) starting immediately after the publication of the first climate change assessment report and as public awareness started to grow (Melillo et al. 1990). A 20 year time-frame has been adopted for other studies in Ethiopia (Deressa et al. 2011) and Uganda (Osbahr et al. 2011). In this study, farm household is the unit of analysis. A ”household” is defined as a farm family unit consisting of a group of interrelated people living together, sharing the same dwelling house, working on the family farm, making farm-level decisions (including adaptation) and pooling their labour to manage their farm under the prime leadership of the household head (Davies and Bennett 2007; Solomon et al. 2007).

Interviews were held with the heads of farm households. The choice of the household head as a primary source of information is justified because the household head plays a primary role in the majority of household and farming decisions related to production, marketing, resource allocation and adaptation decisions in traditional farming (Polson and Spencer 1991; Bryceson 2002; Solomon et al. 2007). However, typically this method does not necessarily capture the direct opinions of all other members of the household and may to some extent be socially biased particularly with respect to activities not carried out by the head of the household. In addition, this study was limited by lack of sufficient observed climate data for the whole of the study period (1992–2012) as long-term historical weather data was difficult to find due to lack of complete data.

### Data collection - methods and tools

This study employed a multiple stage sampling design which involved districts, pastoral/agropastoral associations and farm households. Five out of the seven semi-arid lowland administrative districts of the Borana Plateau were selected for the study in a first stage of sampling (Arero, Dire, Miyo, Moyale, Yabelo). Experts were consulted to ensure that these five districts gave adequate representation of the different agroclimates and farming systems in the region. In the second stage of selection, four associations (two pastoral and two agropastoral) were randomly selected from each of the five districts. At the third stage, 24 households from each of the twenty pastoral/agropastoral associations were then randomly selected to give a total of 480 sample households. The sample size selected was sufficient to allow at least a 95 % confidence level with 5 % precision or margin of error for the parameter estimate in order to draw conclusions from the data analysis (Cochran 1977).

The farm household survey was conducted from August 2012 to October 2012 in two-steps, a field pretest and actual data collection. Enumerators conversant with the local language and customs in the study area were hired to conduct the household interviews during the field research. Before the interviews were carried out, enumerators received field training on the survey instruments and ethical considerations of this research. Each survey questionnaire contained 73 questions and took an average of an hour interview time.

The household survey questionnaire was designed after a review of literature about farmer perception of climate change and its impact on agriculture. The questionnaire was then pretested in the study area to identify potential problems (e.g. unclear questions) and make sure that the questions and methods were tailored to local circumstances. The questionnaire was revised based on feedback from the pretesting through the pilot survey. Research ethics approval was obtained from the Human Research Ethics Committee at the University of Tasmania (Ref# H0012318).

The majority of pastoralists and agropastoralists questioned in the testing phase believed they perceived climate change and that the level of awareness was explained in terms of its impact on local agriculture. A question was therefore added and survey instrument modified to elicit the level of climate change perception through its impact on agriculture using a five-point Likert scale; 1) no perception and thus no perception of impact on agriculture; 2) climate change has been noticeable but has not significantly impacted agriculture; 3) climate change has been noticeable and has impacted agriculture to a certain extent; 4) climate change has been noticeable with a substantial impact on agriculture and 5) climate change has been noticeable and has completely changed the way farming is done. Climate data (monthly rainfall, number of rainy days and monthly temperature), from five meteorological stations covering the study area, were obtained from National Meteorology Agency of Ethiopia. The data were then pooled and mean values were computed to evaluate long-term trends in terms of climate variables.

### Data analysis - the empirical model

A multinomial logistic regression (MNL) modelling approach was used to explore potential relationships between the different levels of smallholders’ perception of climate change and its impact on agriculture (the outcome variable) against a set of household and farm attributes (explanatory variables). It makes use of a general logit transformation which is the logarithm of the odds of a particular outcome level relative to the reference level (Stokes et al. 2000). An MNL can be considered as an extension of a logistic regression where the outcome variable only has two different discrete outcomes. The influences of the explanatory variables in the model on the outcome are summarised using odd ratios (OR) which is the ratio of the odds of an outcome level relative to a reference outcome level (no perceived change).

In the analysis the outcome was the perception level of the climate change and impact of climate change on local agriculture (Stokes et al. 2000; Deressa et al. 2009). It has five levels as detailed above. The first level of ”no perception” of climate change and its impact on agriculture, was used as the reference level. These category levels were used to provide an estimate of a respondent’s beliefs and attitudes on a psychometric scale (Sjöberg 2000b). After exploratory analysis, the second level ‘noticeable but no significant impact on agriculture’ was omitted in the logistic regression analysis as it represented only 1 % of the responses. It is important to point out that the direction and nature of the perceived change, i.e. wetter/drier, better/worse was not captured in the outcome variable, and thus responses with the same outcome value may represent different views by the household heads. However, the overwhelming perception is that perceived changes are not favourable to agricultural production as is reflected by widely perceived reduced rainfall and increased temperature conditions.

The analysis was conducted using Proc Surveylogistic Logistic in SAS Version 9.2 (SAS SAS Institute 2000) where a set of variables initially were considered simultaneously for the analysis. Based on the literature, a set of variables which are relevant to the study area were selected to be included in the list of explanatory variables: household size, livestock holding, farm income, non-farm income, education level, age of the household head, type of production system, access to climate information and extension services. We used Proc Surveylogistic, SAS Version 9.2 (SAS Institute 2000), to ensure that the results were adjusted for the multi-stage sampling. We tested if each variable had a significant simultaneously across all multinomial outcome levels and individually for each multinomial outcome level using Wald chi-square tests. The overall test indicates if an explanatory variable had any effect on an outcome while the individual tests indicate how outcome is effected. For each outcome we also present regression coefficients with the corresponding odds ratios.

Overall model fits was assessed using a Generalized Coefficient of Determination (Cox 1989). Percentage of participants’ responses on climatic variables (rainfall and temperature) were computed using descriptive statistics. The chi-square test was used to test significance of differences between responses, and the responses are summarised as percentages calculated using Proc Surveyfreq, SAS Version 9.2 (SAS Institute 2000). Climatic data were collected for rainfall (amount and number of rainy days), and temperatures for the period prior to (1980–1992), and during (1992–2009) the study period. A regression analysis was then done for three periods (1981–1992; 1992–2009; 1981–2009) to identify any changes in rainfall and temperature in the study area to validate participants’ claim of climate change during the study period and capture potential experiential factors carried forward from the past.

## Results

### Perception of climate change

The majority (96 %) of smallholders interviewed perceived changes in climatic conditions within the twenty-year period between 1992 and 2012 (Table 1). However, 3 % did not perceive any change in climate while the rest (1 %) were unsure of whether the climate had changed or not. Among those who perceived a change, 94 % and 2 % respectively felt there had been a decreasing or increasing pattern, in amount of both seasonal and annual rainfall, over the twenty-year study period. Disaggregation of perception by age - young adults (23–30), adults (31–60) and elderly persons (>61) indicated that young adults are less likely to perceive changes in climatic variables than their older counterparts (Table 1). Meanwhile, disaggregation of perception by production system (pastoral or agropastoral) only revealed significant differences in perception in respect to the direction in which rainfall was changing - both decreasing and increasing.

**Table 1:** Pastoralist/agropastoralists perceptions of existence and direction of changes in overall climate, temperature and rainfall over the past 20 years in the Borana lowlands Ethiopia

Change category	Overall perception	Perception by age group in years				Perception by production system		
		23-30	31-60	61-91	*χ* ^2^ test *P* value	Pastoral	Agropastoral	*χ* ^2^ test *P* value
		% of respondents (n)				% of respondents (n)		
Changes in climate	96(459)	86(37)	97(311)	98(111)	0.00^**^	98(232)	96(227)	0.31^ns^
Increase in temperature	66(312)	46(20)	69(221)	63(71)	0.00^**^	68(162)	63(150)	0.41^ns^
Decrease in temperature	1(6)	0(0)	1(4)	2(2)	0.00^**^	1(1)	2(5)	0.15^ns^
More extremes in temperature	28(132)	37(16)	26(82)	30(34)	0.00^**^	28(67)	27(65)	0.59^ns^
Increase in rainfall	2(10)	0(0)	2(6)	4(4)	0.00^**^	0(0)	4(9)	0.03^*^
Decrease in rainfall	94(448)	86(37)	96(305)	94(106)	0.00^**^	97(230)	92(218)	0.00^**^

Perceptions are subdivided by age and type of production system. Values are presented as a percentage of the group followed by number of respondents in brackets (overall *N* = 475)

Beyond changes in overall climate conditions, smallholders have indicated varying perceptions towards different climatic elements (Table 2). These included increased day and night temperatures with a considerable proportion of them observing more extreme temperature conditions. In terms of rainfall research participants felt there had been a decreasing rainfall amount and shortening duration of the rainy seasons with the majority citing a late onset of the rainy season. It appears that more pastoralists felt overall changes in climate and its elements rather than their agropastoral counterparts.

**Table 2:** Pastoralist/agropastoralists perceptions of existence and direction of changes in temperature and rainfall over the past 20 years (1992–2012) in the Borana lowlands, Ethiopia

Percentage of responses in each category of change (*N* = 475)						
Which direction do you think temperature and rainfall are changing?						
Climatic variable	Climatic element	Increasing	Decreasing	More extremes	Not sure	*χ* ^2^ test *P* value
Temperature (all seasons)	Overall temp	68	1	29	2	0.0001^**^
	Daily temp	95	3	-	2	0.0001^**^
	Nightly temp	57	33	-	10	0.0001^**^
Rainfall (rainy seasons)	Amount	3	95	-	2	0.0001^**^
	Intensity	18	67	-	15	0.0001^**^
How do you see the coming of rains during rainy seasons?						
Rainfall (rainy seasons)	Timing	Early onset	Late onset	More extremes	Not sure	
		1	96	-	3	0.0001^**^
How do you see the length of rainy periods?						
Rainfall (rainy seasons)	Duration	Longer	Shorter	More extremes	Not sure	
		0	97	-	3	0.0001^**^
Which season do you think temperature or rainfall is changing most?						
Changes by season		Long rains	Short dry	Short rains	Long dry	
	Temperature	1	3	94	2	0.0001^**^
	Rainfall	52	0	42	6	0.0001^**^

Values are presented as a percentage of the group followed by confidence limits in brackets (overall *N* = 475)

Figures in brackets show lower and upper confidence limits at 95 % confidence level

Study participants who perceived change in climate were concerned about the magnitude and direction of change emphasising that they had the impression of a worsening climate unfavourable for local agriculture (58 %), a more unpredictable climate (29 %) and more extreme weather events (5 %). Only a minority of 8 % of the respondents thought that the climate was becoming more favourable for agricultural production. Asked about the likely future of their livelihoods in ten years, 40 % of respondents answered that they would be worse off, 27 % better-off and 26 % found the future difficult to predict due to uncertainty in future climate. Only 7 % of the interviewees believed that their livelihoods would remain unchanged. However, all respondents agreed that their future relies on how well climate favours their livestock dominated rainfed production system.

### Perceived level of climate change and its impact on agriculture

**Fig. 2:**
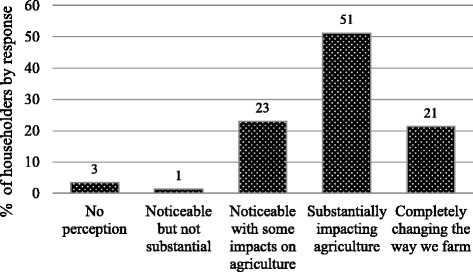
Climate change as perceived in terms of its impact on agriculture over the period 1992-2012 by farm households (pastoralists/agropastoralists) in the Borana lowlands, Ethiopia

Pastoralists and agropastoralists expressed their perception of climate change in terms of its impact on agriculture to varying degrees (Fig. 2). In total, only 3 % of respondents did not perceive any change in climate over the study period, 1992 to 2012, while 96 % of them believed the impact of climate change was noticeable with varying degrees of impact on their traditional agriculture.

While nearly all of the participants perceived climate change to some extent only 70 % had access and were informed by up-to-date climate information, 80 % got access to extension service and average age of the participants is close to 50 years (Table 3). All smallholders associated the impact of climate change with reductions in crop/livestock production and considered such reductions as a salient risk posed to their agriculture-dependent livelihoods. While smallholders predominantly depend on subsistence agriculture for their livelihood, income from non-farm activities contribute nearly a third of the total income (Table 3).

**Table 3:** Description of variables used to create a logistic regression model for climate change perception of pastoralist/agropastoralists in the Borana lowlands, Ethiopia

(a) Outcome variable	Description	Respondents who perceived % (score values)	Respondents who did not perceive % (score values)
Level of climate change and its impact on agriculture perceived over the past 20 years	Takes the scores from 0 (no perception) to 4 (Noticeable and completely changing the way we farm)	96 (1–4)	4 (0)
(b) Explanatory variables		Mean	SD
Age	Age of the HH head in years; categorical, 1 if young adult, 2 if adult, 3 if old	49.8	15.3
Education	School attendance; categorical, 1 if no formal education, 2 if primary, and 3 if secondary	1.1	2.6
Household size	Family size of the household in heads; continuous	7.4	2.8
Production system	Production system dummy; 0 if agropastoral and 1 if pastoral	0.5	0.5
Livestock holding	Herd size of the HH in TLU^a^; continuous	8.1	9.6
Farm income	Annual farm income in USD; continuous	461.9	364.1
Non-farm income	Annual non-farm income in USD; continuous	219.7	375.6
Access to climate information	Access to climate information dummy; 1 if yes otherwise 0	0.7	0.5
Access to extension service	Access to extension service dummy; 1 if yes otherwise 0	0.8	0.4

^a^TLU = Tropical Livestock Unit; HH = Household, $US =18 Ethiopian Birr in 2012

The MNL analysis indicated that effects of age, level of education, livestock holding, access to climate information and access to extension services in determining perception levels were found statistically highly significant (P < 0.01) (Table 4). Increased odds of perception was associated with increased age (2 vs 3), level of education (1 vs 3), livestock holding, access to climate information and extension services. However, the variables household size, farm and non-farm incomes and production systems were found non-significant in terms of predicting perception levels. Moreover, the three perception levels have responded to varying odds to different explanatory variables used in the analysis. In summary, household size, farm and non-farm incomes and production systems were not important predictors of perception levels of smallholders.

**Table 4:** Parameter estimates for the full model and marginal effects from the multinomial logistic regression model of the perceived degree of climate change and its impact on agriculture over the last 20 years by farm households in the Borana lowlands

Explanatory variable	Overall significance of the variable (*P*-value)	Groups	Noticeable and having some effects on agriculture (*N* = 109)		Noticeable and substantially affecting agriculture (*N* = 243)		Noticeable and completely changing the way we farm (*N* = 101)	
			Coefficient (Odds ratio)	*P*-value	Coefficient (Odds ratio)	*P*-value	Coefficient (Odds ratio)	*P*-value
Age	0.000^**^	Age 1 vs 3	−2.130(0.035)	0.000^**^	−0.964(0.167)	0.069^ns^	−1.563(0.061)	0.006^**^
		Age 2 vs 3	0.900(0.717)	0.020^*^	0.137(0.502)	0.741^ns^	0.326(0.402)	0.391^**^
Education	0.008^**^	Cat 1 vs 3	0.399(0.900)	0.515^ns^	0.597(2.299)	0.294^ns^	1.002(3.029)	0.091^ns^
		Cat 2 vs 3	−0.905(0.244)	0.146^ns^	−0.361(0.882)	0.561^ns^	−0.875(0.454)	0.173^ns^
Household size	0.143^ns^	–	0.015(1.015)	0.923^ns^	0.065(1.067)	0.643^ns^	0.103(1.108)	0.472^ns^
Production system	0.566^ns^	–	−0.047(0.954)	0.936^ns^	−0.289(0.749)	0.595^ns^	0.029(1.029)	0.963^ns^
Livestock holding	0.004^**^	–	0.436(1.547)	0.001^**^	0.441(1.554)	0.001^**^	0.399(1.492)	0.001^**^
Farm income	0.086^ns^	–	−0.0004(1.000)	0.767^ns^	−0.0002(1.000)	0.869^ns^	−0.0023(0.998)	0.172^ns^
Non-farm income	0.159^ns^	–	0.0009(1.001)	0.474^ns^	0.0016(1.002)	0.160^ns^	0.0015(1.001)	0.185^ns^
Access to climate information	0.001^**^	–	1.750(5.751)	0.007^**^	1.990(7.313)	0.001^**^	1.351(3.861)	0.038^*^
Access to extension service	0.001^**^	–	1.556(4.742)	0.033^*^	1.786(5.963)	0.002^**^	0.824(2.278)	0.247^ns^
Observations used	465							

reference level = no perception of climate change; pseudo-R^2^ = 0.223; ** = highly significant; * = significant; ns = non-significant

Different levels of perception were found to have shown different odds for a unit change in various explanatory variables (Table 4). The odds of perception at all levels increased significantly as livestock holding increased by an additional TLU. Access to support services also improved likelihood of perception among smallholders. Smallholders’ access to climate information was highly significantly associated with increased odds of perception at all levels as compared to those who did not perceive. In addition, advisory support through extension services has significantly improved odds of perception at the lower and middle level, albeit non-significantly at the highest level. The likelihood for higher levels of perception were significantly increased with increasing household size though not statistically significant.

### Perceived causes of climate change

When asked about primary causes of climate change, no one mentioned the role of greenhouse gases in driving climate change. Supernatural forces (45 %), natural (physical) process (33 %) and deforestation due to human action (16 %) were mentioned as major drivers of climate change. A small number, 6 %, of respondents were unsure or could not give an explanation for what the cause for climate change could be.

### Climate data for 1992–2012 and 1980–1992

Climate data indicate that temperature has shown a significantly increasing trend (P < 0.05) for the 1992–2009 period (Table 5). However, increasing trend in rainfall amount was observed during the study period across the two rainy seasons though the one in the long rains season was not statistically significant. Simultaneously, the number of rainy days highly significantly decreased (P < 0.01) during the long rains season while the change was not significant for the short rains season of the 1992–2009 period. In contrast to trends in the study period, the preceding period 1981–1992 was characterized by a sharp decline for rainfall amount across the two rainy seasons which might have carried an experiential legacy among participants. Number of rainy days and temperature did not however show any significant trends during same preceding period.

**Table 5:** Changes in the moving averages of observed climatic variables between 1981 to 2009 in the Borana pastoral/agropastoral systems, Ethiopia

Season	Duration	Rainfall		Number of rainy days		Air temperature	
		Slope(mm/year) (R^2^)	P-value	Slope(days/year) (R^2^)	P-value	Slope (°C/year) (R^2^)	P-value
Long rains	1981-1992	−26.97 (0.65)	0.001	−1.46 (0.28)	0.092	−0.07 (0.13)	0.241
	1992-2009	+1.97 (0.10)	0.190	−0.43 (0.37)	0.009	+0.02 (0.32)	0.013
	1981-2009	−9.10 (0.50)	0.000	−0.64 (0.45)	0.000	+0.01 (0.01)	0.732
Short rains	1981-1992	−8.83 (0.63)	0.002	+0.03 (0.00)	0.909	+0.17 (0.19)	0.154
	1992-2009	+4.76 (0.25)	0.034	+0.05 (0.01)	0.758	+0.04 (0.32)	0.014
	1981-2009	+0.73 (0.02)	0.469	+0.13 (0.12)	0.072	+0.05 (0.17)	0.024

## Discussion

### Perception of climate change

The large majority of respondents in the study area believed to have experienced climate change during the study period. Study participants indicated that they perceived changes in temperature and rainfall, expressed mainly in terms of patterns in weather experienced; higher temperatures, below normal rainfalls and short rainy seasons, higher frequency and intensity of extreme weather events. Similar expressions of awareness by farmers about climate change have been reported in studies conducted over the same two decades as this study in Ethiopia (Legesse et al. 2013), Nigeria (Tambo and Abdoulaye, 2013) and Chile (Roco et al. 2014). Except in the case of study from Chile, no attempt was made in these studies to relate perceptions of climate change to actual climatic data from meteorological sources.

During the 20-year period covered in the survey in this study (1992–2012) the limited meteorological evidence suggests that climatic change in terms of seasonal temperature did occur during that period. In addition, a significantly decreasing rainfall amount during short rain season and increasing number of rainy days during the long rain season was observed. The observation that number of rainy days slightly increased in the short rainy season may suggest the shift of rainy days from long into short rainy seasons in the study area. However, the claim by majority of respondents, rainfall amount has declined, cannot be substantiated by meteorological evidence and is contrary in statistical terms. There might be other attributes of the actual climate in the area that have not been captured in this dataset, which potentially contributed to shape smallholders’ perception of climate change.

**Fig. 3:**
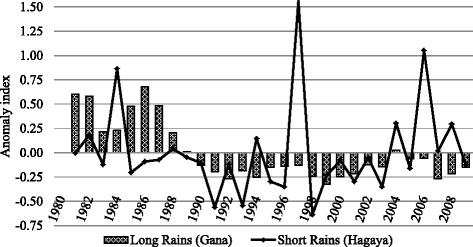
Rainfall anomaly indices for the two rainy seasons in the Borana lowlands between 1981 and 2009 (Data source: NMA, 2012)

Although this data should be treated with caution, the apparent minimal changes during the study period means a valid area-wide perception would be that climate change had occurred over the 20 year period. However during the two decades of the study (1992–2012) the rainfall was less, there were fewer rainy days and higher temperatures in comparison to the preceding decade (1980–1992) (Fig 3). This indicates that if the respondents were comparing the two phases (1980/1992 to 1992/2012) their perceptions were clearly based in reality and experience. Thus within this study and in spite of the clear and explicit instructions only to consider the past 20 years, it is highly likely that perceptions were primarily modified by experiential factors especially perception of change from an earlier period.

Other factors modifying perception may be genuine localised change, increased climate variability mainly extremes or a complex of interacting climatic and non-climatic forces. Such factors were simplified in perception to be the result of climate change. In particular, recent experience of worsening circumstances under periodic and severe drought (e.g. in 2008 and 2011) may drive a belief in a climate that has significantly changed during the study period. Whitmarsh (2008) argues to the human tendency to readily trust “their interpretation of an experience” as reality and as such causal attribution may not be well-managed (Ahn et al. 1995). However, this perception still remains relevant to climate change related policy and decision-making as it prompts smallholders for adaptive action and their willing cooperation with others.

The lesser perception of climate change by younger householders (86 % vs 98 %; Table 1) may reflect less exposure to climate stimuli and reduced experience in terms of dealing with changing farm conditions and activities. This lesser perception by younger householders agrees with findings of similar studies conducted in farming systems from semi-arid Africa (Deressa et al. 2011; Silvestri et al. 2012; Teka et al. 2013). However, the discrepancy between young households noticing climate change and the lack of continuous and complete meteorological evidence for the study period might be attributed to an influence from their older counterparts citing that climate was better in the previous decades.

Findings suggest that more pastoralists were pessimistic than optimistic about the future of their farming livelihoods. Similar study by Yu et al. (2013) reported that majority of the public discern that climate change does harm to local residents and society. The Boran smallholders’ experience during the study period (1992–2012) with rainfall conditions consistently below those in the preceding decade had been traumatic, threatening their climate-sensitive agriculture-dependent livelihoods. Under limited access to climate information, perception of the magnitude and direction of climate change is often explained in terms of beneficial or adverse changes in the livelihood of rural communities (Gandure et al. 2013). Vulnerability to drought appears the major factor driving perception of climate change and salient risk to local agriculture and livelihoods.

### Perceived level of climate change and its impact on agriculture

The data analysis results suggested that various household and farm attributes affected perception of rural smallholders about climate change and its impact on local agriculture. The farm income of larger herders has been observed to be more sensitive to adverse climate change (Seo and Mendelsohn 2007). This sensitivity was observed in this study. An additional unit of livestock (TLU) on average increased the odds of feeling limited effects of climate change by a factor of 1.520 as compared to those farm households who did not perceive any changes (Table 4). Livestock keepers with a cattle dominated herd structure in particular find it difficult to cope up with feed shortage during drought years (Lesnoff et al. 2012).

This study suggests that educational activities - years of school, access to climate information and extension services strongly influence and increase perception of climate change and its impact on local agriculture. A higher level of education may slightly result in a greater awareness of climate change as a real issue of global and immediate concern, thus increasing the likelihood that changes in farming practices are attributed to the impact of climate change. However, the odds of increasing likelihood of the perception levels is not statistically significant which might be attributed to the observation that more educated ones are the younger ones. Smallholders who are more educated are more likely to be able to interpret and apply climate information to their lives making them aware of local climate change or variability which becomes crystallised into a perception of climate change. The impact of education and access to weather information (i.e. a higher level of education is associated with a greater probability of perception of climate change) is common among smallholder farmers across African farming systems (Mustapha et al. 2012; Ndambiri et al. 2012; Amdu et al. 2013). Studies in Kenya, Ghana and Zimbabwe indicate the significant benefits that accrue from local climate information (e.g. weather updates) which increase the awareness of climate change in terms of more informed adaptive decisions and improved technology uptake among smallholder farmers (Kalungu et al. 2013; Mapfumo et al. 2013).

Income (farm or off-farm) does not significantly modify smallholders’ perceived level of climate change and its impact on local agriculture. Results indicated that farm households in the study area on average earn nearly a third (32 %) of their annual income from non-farm sources and dependency of income is therefore associated with external contingencies influencing off-farm income generating activities or employment and does not drive a perception of climate change. Off-farm income will also increase diversification opportunities and therefore buffer any income losses and adverse effects of climatic events such as drought.

In the regression analysis result (Table 4), livestock holding was seen to be associated with climate change perception. This may be driven by the reliance of livestock on pasture and water, which are both climate-sensitive resources. Access to advisory support through extension services also improved perception of farming communities. This can be attributed to the advisory support and training programs that assists smallholders to take climate change as problem and context which need to adapt to. In addition, production system did not matter to affect smallholders’ perception of climate change which might be attributed to the shared high level of vulnerability of rainfed farms to climatic shocks. In general, these variables appear to be important factors to work on in order to influence perception of local communities and ensure their willing participation in decision-making at different scales which can be translated into useful policy information.

### Perception of causes of climate change

Like many traditional communities in sub-Saharan Africa, a significant proportion of Boran smallholders (45 %) consider that humanity is cursed and supernatural forces are the primary cause of climate change. Disobedience and unfaithfulness to God’s rules, failure to glorify him and divergence from the age-old Boran tradition have led to divine punishment, especially drought events. This spiritual perspective is widespread in Africa (Patt and Schröter 2008; Gandure et al. 2013; Tambo and Abdoulaye 2013). Similarly, Teka et al. (2013) reported farmers in Benin partly attributed climate variation to failure in observance of traditional customs and endogenous laws by the indigenous community.

Others (33 %) freely acknowledge that climate is changing but do not associate climate change with human activities except for deforestation. While traditional smallholder farmers would not be exposed to information about greenhouse gas emissions (which for a rural traditional farmer would be an intangible and complex concept), they would have directly experienced that indiscriminate tree cutting for firewood and charcoal production aggravates desertification (Nyanga et al. 2011). A small proportion of interviewees (6 %) were not sure or have no idea about the drivers of climate change perhaps reflecting the complex and intangible nature of climate change.

## Conclusion

Smallholders in Borana pastoral/agropastoral systems overwhelmingly had a perception of a changing climate between 1992–2012 although there is limited meteorological evidence of significant change during that period. It is highly likely that perception was primarily modified by other factors such as experiential factors especially perception of more rainfall in the 1980s and the severe droughts experienced recently including in 2008 and 2011. The later extreme events may explain the overwhelming pessimism by farmers about the future of their livelihoods and their view that climate change will increasingly and negatively impact agriculture. Extreme events in this regard play crucial role in influencing participants’ attitude.

While perception of climate change by smallholders does not seem to relate to the direction to which climate variables actually change, it is related to access to climate information. Smallholders do have varying levels of perception and attitudes towards climate change and its impact which are interwined with non-climatic forces pervasive social, economic and political changes. Due to smallholders’ sensitivity to an inherent climate variation, this is likely to interact with poor attribution to other potentially confounding factors. A lack of perception may risk an unnecessary transition to cultivation and non-pastoral livelihoods.

Pastoral and agropastoral communities have not been actively engaged in the national policy landscape and this has been partly attributed to a biased approach towards modern agriculture and negative attitude towards pastoralism often seen as a waning lifestyle. This attitude has resulted in a poor understanding of development needs and priorities for these farming communities living at the edge increasingly constrained by climate change. Pastoralists, agropastoraists and their communities are clearly aware of climate change and variability and are very pessimistic about the potential impact on their livelihoods into the future. This is very critical to gather willing cooperation of the intended beneficiaries in adaptation programs and policy making which prompts need for adaptation. Moreover, it is crucial to enhance their understanding of risks associated with climate change so that they have realistic expectations and are better prepared not only for the potential negative impacts but also for taking advantages of any opportunities climate change offers.
